# Intra-Articular Administration of PBHSCs CD34+ as an Effective Modality of Treatment and Improving the Quality of Life in Patients with Coxarthrosis

**DOI:** 10.3390/jcm14082656

**Published:** 2025-04-12

**Authors:** Marek Krochmalski, Marek Kiljański, Jakub Krochmalski, Piotr Grzelak, Karolina Kamecka, Mariusz Mianowany, Jarosław Fabiś

**Affiliations:** 1 Orthopedic Surgery Department, Outpatient Orthopedic Care, Medical Magnus Clinic, 90-552 Lodz, Poland; 2 Polish Muscles, Ligaments and Tendons Society, 90-552 Lodz, Poland; 3 Rehabilitation Department, Medical Magnus Clinic, 90-552 Lodz, Poland; 4 Polish Association of Physiotherapy Specialists, 95-200 Pabianice, Poland; 5 Imaging Diagnostics Department, Medical Magnus Clinic, 90-552 Lodz, Poland; 6 Department of Diagnostic Imaging, Polish Mother’s Memorial Hospital—Research Institute, 93-338 Lodz, Poland; 7 Department of Physiology, Pathophysiology and Clinical Immunology, Medical University of Lodz, 90-419 Lodz, Poland; 8 Department of Arthroscopy, Minimally Invasive Surgery and Sports Traumatology, Medical University of Lodz, 90-419 Lodz, Poland

**Keywords:** osteoarthritis, coxarthrosis, total hip prosthesis, stem cells, CD34+/CD34 positive, peripheral blood HPSCs, progenitor cells, hematopoietic stem cells, HOOS, SF-36

## Abstract

**Background/Objectives:** In 2020, 595 million world citizens had osteoarthritis, and the largest growth in OA morbidity refers to the hip joint. Effective OA therapies have been sought for years. Assessing the treatment effectiveness and QoL improvement in hip OA after intra-articular administration of fresh peripheral blood hematopoietic CD34+ stem cells. **Methods:** The study comprised 49 adults (median age: 63). The SCs were injected into hip joints and straight to the bone. Hip manipulation was conducted. Patients were subjected to a standardized rehabilitation protocol. Hip degeneration was graded by Kellgren–Lawrence. Multi-factor statistical analyses, with replications, were performed. The study was an R&D project, co-financed by the E.U. **Results:** Patient-reported outcomes (HOOS, SF-36) ameliorated remarkably over 24 months (*p* < 0.0001). Ranges of movement improved significantly (*p* < 0.0001). The most noticeable improvement manifested 6 months after the SC administration. Its furtherance was maintained. **Conclusions:** Intra-articular administration of CD34+ cells significantly reduces pain and improves hip joint function, regardless of the severity of OA, according to K-L, over a 24-month follow-up period. The combination of CD34+ cell therapy with joint mobilization and rehabilitation allows for the postponement of hip arthroplasty by significantly improving patients’ QoL over the 24-month follow-up period.

## 1. Introduction

Coxarthrosis (hip osteoarthritis, hip OA) is a degenerative joint disease significantly impacting the quality of life (QoL) of affected individuals, leading to pain, decreased mobility, and functional impairment. Global studies indicate that 595 million people had osteoarthritis (OA) in 2020, which equals 7.6% of the world population [1]. This massive worldwide burden of OA is projected to increase significantly by 2050, with the largest growth anticipated for the hip joint, estimated at 78.6%. Even though the problem of growing incidence has been widely announced for years, the search for the “ideal” therapy still continues [2,3,4,5,6,7,8,9]. Current treatment strategies for hip OA involve conservative methods such as physical therapy, analgesics, and intra-articular injections of collagen and hyaluronic acid, and also biological methods such as platelet-rich plasma (PRP), platelet-rich fibrin (PRF) and stem cells injections. Surgical interventions such as total hip arthroplasty remain effective but carry inherent risks, are costly procedures often requiring even more expensive and difficult revisions and are typically reserved for advanced cases [10,11].

Is it an unstoppable chronic and destructive disease? The severity of symptoms caused by OA and their impact on humans was expressed in meaningful words given by an outstanding Polish orthopedist and founder of rehabilitation in Poland, Prof. Wiktor Dega, who said the following: Sick hip–sick men [12]. This statement encourages the undertaking of intensified and ongoing actions toward hip OA prevention and effective therapies.

Prof. Arnold Caplan noted that cell-based therapy is in its early stages, requiring researchers to adapt their methods as new information emerges. He emphasized that critical discussions are also helping to improve the effectiveness of these therapies [13,14,15].

With an aging global population and expecting a rise in the incidence of coxarthrosis, it is necessary to develop effective treatment modalities that can address both symptom management and disease progression.

In recent years, regenerative medicine has gained traction as a novel therapeutic avenue for managing OA, particularly through the use of stem cell therapy. The stem cells possess unique properties, including the ability to differentiate into various cell types, secrete growth factors, and modulate inflammatory responses, making them an attractive option for cartilage repair and regeneration. Among the various stem cell types, CD34+ hematopoietic stem cells derived from peripheral blood (PBHSCs CD34+) have demonstrated significant potential in preclinical and clinical studies [16]. These cells are known to have regenerative capabilities that can potentially restore joint function and improve patient outcomes. The intra-articular administration of PBHSCs CD34+ targets the underlying mechanisms of coxarthrosis by promoting tissue repair and reducing inflammation. Studies have shown that these cells can enhance cartilage regeneration, improve synovial fluid composition, and reduce pain levels, thereby improving the overall quality of life in patients [17,18]. However, despite promising preliminary findings, the clinical application of CD34+ stem cells for coxarthrosis remains underexplored, with a need for standardized treatment protocols and long-term efficacy data.

Regardless of hip arthroplasty, which definitively resolves the clinical problem of osteoarthritic changes, alternative treatment methods continue to be explored, even if they provide only temporary improvement. Numerous clinical studies have demonstrated the efficacy of intra-articular administration of saline, hyaluronic acid, platelet-rich plasma (PRP), bone marrow stem cells (BMSCs), and adipose-derived stem cells (ADCs) in reducing pain and improving hip joint function in osteoarthritis of varying severity [19,20,21,22,23,24,25]. Considering these findings, we conducted our own study to evaluate the effectiveness of intra-articular injections of a phenotypically well-defined population of peripheral blood CD34+ cells in combination with joint mobilization and a standardized rehabilitation protocol [26].

Therefore, the goal of this study was to assess and analyze the effectiveness of the implemented treatment modality and improvement in health-related quality of life in patients with coxarthrosis after intra-articular administration of PBHSCs CD34+. The article aims to analyze patient-reported outcomes related to pain relief, joint functionality, and overall quality of life. Based on our interdisciplinary clinical and research team experience, we propose standardized holistic therapy using the body’s own forces to significantly slow down or even stop OA development. Additionally, the authors will address challenges and limitations associated with this treatment modality, proposing future directions for research in this promising area of regenerative medicine.

## 2. Materials and Methods

### 2.1. Procedure

The study comprised 49 adults. The fresh PBHSCs CD34+ obtained by apheresis from peripheral blood were injected in the operating theater under lumbar anesthesia and US guidance to 95 hip joints and straight to the bone in case of a soft femur head. Hip joint manipulation was conducted. The study participants were subsequently subjected to a standardized rehabilitation protocol. The study participants’ median age was 63 years. According to the Kellgren–Lawrence Grading Scale, 1st degree degeneration was found in 20.31% of hip joints, 2nd degree in 35.94%, 3rd degree in 37.50%, 4th degree in 6.25%. The study was conducted within a research and development project co-financed by the E.U. (project co-financing agreement number RPLD.01.02.02-10-0125/19-00 under Priority Axis I: Research, development, and commercialization of knowledge of the Regional Operational Program of the Łódź Voivodeship for the years 2014–2020) in The Medical Magnus Clinic, which is an inpatient and outpatient medical center specializing in orthopedics and rehabilitation of the musculoskeletal system.

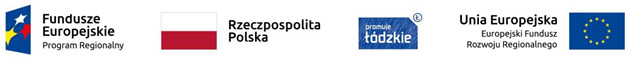



The protocols of this study were approved by the Bioethics Committee of the Medical University of Lodz, Poland (resolution No. RNN/19/22/KE, 12 April 2022). All participants were fully informed and signed an individual consent form for participation in the study.

The project encompassed the following stages:

1. Qualification based on clinical and MRI and X-ray examinations;
2. Collection of PHSCs CD34+ through apheresis;
3. Phenotypic and quantitative assessment of the stem cells obtained;
4. Intra-articular injections of PHSCs CD34+ within the operating theater;
5. Rehabilitation during hospital stay at the Regenerative Medicine Department;
6. Rehabilitation on an outpatient basis;
7. Periodic medical and physiotherapeutic checkups after 6 weeks, 3 months, 6 months, 12 months, and 24 months; MRI after 6 months, 1 year, and 2 years; and X-ray after 2 years;
8. Analysis and evaluation of the results;
9. Introduction of a novel medical procedure into the health market in the case of its proven efficacy;
10. Scientific publishing process.

### 2.2. Methods

Obligatory procedures before administering the stem cells consisted of the following:

- Patient functional assessment;
- Mobility assessment of the hip joints (angular measurements).

The cells were obtained by apheresis from patients’ peripheral blood using a dedicated program for CD34+ cells, just as cells are collected during leukapheresis (Spectra Optia™ Apheresis System, Terumo BCT, Inc., Lakewood, CO, USA). The obtained cells were not subjected to any processing and after their collection, they were administered by intra-articular injection no later than 4 h after their collection. The number of cells was counted by cytometry (BD FACSLyric^TM^, BD Biosciences, San Jose, CA, USA). The cells were not previously stimulated to increase their number.

In the beginning, we injected joint local anesthesia without any correction of ROM. During periodic examinations, it was noticed that the joint was moving like “a cog in a machine”. The next step was intravenous short anesthesia and, finally, spinal anesthesia (all the patients were adults).

In order to assess long-term patient-relevant outcomes following hip injury, the Hip Disability and Osteoarthritis Outcome Score (HOOS) questionnaire was employed. The HOOS is user-friendly and self-administered. It encompasses five important outcomes: pain, symptoms, activities of daily living, sport and recreation function, and knee-related quality of life. The HOOS meets the basic criteria of outcome measures and can be used to evaluate the course of knee injury and treatment outcomes [27,28].

When evaluating the broadly understood quality of life, America’s SF-36 Health Survey was adapted and used thereafter. The authors used the Polish-language version of the questionnaire. The relevant results showed that the factor structure of the Polish version of SF-36v2 differed from its original American version. The Polish translators and researchers had decided to maintain the original subscales in the Polish version of SF-36v2 in order to provide the means for future comparisons between data acquired in different countries. According to the Polish research team, there had been reasonable indicators of validity and reliability of the Polish version of SF-36v2 supporting their final decision.

The research tool comprises 36 questions that cover eight domains of health:

1. Limitations in physical activities because of health problems;
2. Limitations in social activities because of physical or emotional problems;
3. Limitations in usual role activities because of physical health problems;
4. Bodily pain;
5. General mental health (psychological distress and well-being);
6. Limitations in usual role activities because of emotional problems;
7. Vitality (energy and fatigue);
8. General health perceptions.

Patients were asked to fill out the questionnaire by themselves. Afterward, it was scored by a local data manager and statistician [29,30,31].

Measurements of the range of motion in the hip joint were performed in accordance with the SFTR measurement and recording standard. The source of the SFTR abbreviation is the first letters defining the appropriate planes in which the movements are tested, i.e., S—sagittal, F—frontal, T—transverse, and R—rotation. The measurements are recorded using three digits according to the following rules: (1) the first to be recorded are extension movements, all movements carried out from the body, external rotation of the limbs, abduction, inversion, and when examining the spine, side bends, and turns to the left; (2) the middle digit represents the starting position, in physiological conditions it is usually 0; and (3) the last digit covers flexion movements and movements toward the body, internal rotation of the limbs, adduction, pronation, side bends, and turns to the right when examining the spine. Movements at all joints were measured from a neutral zero starting position. To check the range of motion for a specific joint, the goniometer was placed at a specific point, and the patient was asked to perform a given movement. Then, the movable arm of the goniometer was employed in order to read the result. The ranges of motion in the frontal plane and rotational movements are performed in a supine position on the back. However, movements in the sagittal plane, flexion, and extension are performed while lying sideways on the untested side. It should be mentioned that the SFTR modality enables the researcher to examine both active and passive ranges of motion. Active refers to the ability to move an extremity independently during movements. As a result, it provides the physiotherapist with information regarding the quality and fluidity of movements, and the patient’s ability to move the limb. Additionally, the active measurements make it possible to determine whether the patient has any mobility limitations. Passive refers to the movement in a joint that is achieved by the researcher while moving a segment without the patient’s involvement [32,33].

Muscular strength is defined as the ability to exert a force on an object or against an external resistance. The apt assessment of muscle strength makes it possible to set precise rehabilitation or training goals, and to evaluate fluctuations over a period of time. During the study, the muscle strength in the subjects’ lower extremities was measured in vivo with the use of a K-Force Muscle Controller (Kinvent Hellas, Thessaloniki, Greece). According to the manufacturer, the K-Force Muscle Controller is a handy dynamometer designed for the measurements of muscle strength. It enables the researcher to assess isometric strength, both the peak force and average force of a specific muscle or muscle group. The dynamometer also allows for measuring the muscle strength together with its deficit due to injuries and comparing them to the strength of the unaffected side. The dynamometer has become the “gold standard” because it carries out undisputably precise measurements of the strength of a muscle or a muscle group of a patient. It also allows the researcher to objectify improvement after the implementation of a protocol. The dynamometers make it possible to obtain a genuine value of the muscle force (in Newtons, Kilograms Force, or pounds) that is exerted on them during the procedure. Kinvent tools enable precise and easy assessment and data flow in a digital format. During the procedure, the study participants were asked to exert maximum effort during an isometric contraction to appraise isometric strength. The researcher stabilized the dynamometer in a fixed position while the subject applied maximal effort against it, gradually increasing the contraction for one second in order to avoid position and stabilization errors and to “push as hard as you can” for five seconds. The subjects were instructed to carry out three consecutive contractions with maximal effort with their dominant limb, each lasting five seconds and followed by a short period of 30 s of rest between repetitions, aimed at avoiding fatigue. The participants had undergone spot-on training and acquired instructions on how to use the dynamometer prior to data collection. The training session was conducted by an experienced practitioner with more than 20 years as a physiotherapist and who had worked with the Kinvent for 2 years [34,35,36,37,38].

### 2.3. Statistical Procedures

The normality of numerical traits was tested by using the Shapiro–Wilk W test. A multi-factor analysis of variance (ANOVA) with repeated measures was performed to assess the dynamics of the studied outcomes together with between-group differences for normally distributed numerical traits throughout the observation period, at baseline (before implanting the stem cell) 6 weeks, 3 months, 6 months, 12 months, and 24 months and after administering the stem cells. Generalized estimating equations (GEEs) were fitted for non-normally distributed numerical variables. A *p* value < 0.05 was deemed statistically significant. The statistical computations were carried out using Statistica™, release 13.3 (TIBCO Software Inc., Palo Alto, CA, USA).

## 3. Results

### Characteristics of the Study Group

The present study comprised 49 adults: 18 were women (36.73%), and 31 were men (63.27%). In this cohort, stem cells were injected, in total, into 95 hip joints. In the studied group of patients, the cell content in the 40 mL suspension administered intra-articularly ranged from 140,000 to 2,712,000 (M = 1,019,500; SD +/− 649,912). Among the study participants, 72 (75.79%) were procedure naïve (the stem cells were administrated to them for the first time), while 23 (24.21%) underwent it for the second time. Both subcohorts were henceforth named Group One and Group Two, respectively.

The mean age of the study subjects was 61.05 years (SD 11.34 years). Complementarily, the median and mode values amounted to 63 and 65 years, respectively. Their BMI (Body Mass Index) averaged 28.40 kg∗m^−2^ (SD 4.46 kg∗m^−2^). For completeness’ sake, 54.66% of the study participants were overweight, 26.67% suffered from obesity, and only 18.67% had a normal body mass (Table 1).

In order to appraise patients’ symptoms and functional limitations along with subjective opinions about their hip-related quality of life during a therapeutic process, the Hip disability and Osteoarthritis Outcome Score (HOOS) was used.

The pain severity changed significantly in both study subcohorts over 24 months of follow-up (respectively, *p* < 0.0001). The relative change was 9.27% in plus in Group One (improved) and 7.12% in minus in Group Two (deteriorated). The multi-factor model showed that the dynamics of the phenomenon did not differ statistically significantly between them (*p* = 0.1459). However, it is necessary to pay attention to statistically significant discrepancies in the assessment of pain intensity that emerged between the study subcohorts at baseline (*p* = 0.0001). On average, the participants who were SC naïve rated the pain severity at 74.27% (SD 23.48%), while the subjects who had received SC earlier assessed the pain severity at 62.39% (SD 21.42%). The relative difference was 19.04% in favor of Group One. In Group One, the most marked improvement was noticed 6 months after the SC administration (by 9.34%). It should be emphasized that the furtherance was maintained until the end of the program (Table 2).

The symptoms and stiffness changed significantly in both study subcohorts over 24 months (respectively, *p* < 0.0001 and *p* = 0.0004). The relative change was 7.98% in plus in Group One and 4.44% in minus in Group Two. The dynamics differed statistically significantly between them (*p* = 0.0324). Similarly, a statistically significant difference in the baseline evaluation of symptoms and stiffness was observed between the study groups (*p* = 0.0004). On average, the symptoms and stiffness were assessed at 71.04% (SD 23.13%) in Group One and 55.65% (SD 21.12%) in Group Two. The relative difference was 27.65% in favor of SC naïve participants. In Group One, the largest benefit was achieved 6 months after the procedure (by 6.35%). It was maintained, even slightly increased, until the end of the Project (Table 3).

The appraisal of quotidian activities changed significantly in both study subcohorts over 24 months (respectively, *p* < 0.0001 and *p* = 0.0002). The relative change was 8.75% in plus in Group One and 4.97% in minus in Group Two. The dynamics did not vary significantly between them (*p* = 0.1872). At baseline, a significant between-group difference was observed (*p* = 0.0003). On average, the activities of daily living were rated at 73.45% (SD 24.15%) in Group One and 63.17% (SD 21.19%) in Group Two. The relative difference was 16.27% in favor of Group One. In Group One, the largest improvement was noticed 6 months after the procedure (by 10.74%). It was preserved until the end of the program (Table 4).

The assessment of sports and recreational activities changed significantly in both study subcohorts over 24 months (respectively, *p* < 0.0001 and *p* = 0.0176). The relative change was 7.94% in plus in Group One and 13.17% in minus in Group Two. The dynamics were not significantly different in both subcohorts (*p* = 0.0784). At baseline, a statistically significant between-group difference was found (*p* = 0.0003). On average, the sports and recreational activities were assessed at 66.23% (SD 28.10%) in Group One and 49.73% (SD 25.53%) in Group Two. The relative difference was 33.18% in favor of Group One. In Group One, the largest boost was noticed 6 months after the procedure (by 9.26%). It was maintained until the end of the program (Table 5).

The evaluation of hip-related quality of life changed significantly in both study subcohorts over 24 months (respectively, *p* < 0.0001 and *p* = 0.0015). The relative change was 10.55% in plus in Group One and 15.55% in minus in Group Two. The dynamics did not significantly differ between them (*p* = 0.1104). At baseline, a statistically significant between-group difference was observed (*p* = 0.0021). On average, the sports and recreational activities were assessed at 59.98% (SD 25.23%) in Group One and 42.39% (SD 26.45%) in Group Two. The relative difference was 41.50% in favor of Group One. In Group One, the most marked improvement was noticed 12 months after the administration of SC (by 15.01%). It was preserved until the end of the program (Table 6).

The pooled range of movement in the subjects’ hip joints changed significantly only in Group One (*p* = 0.0476). The relative change was 21.35% in plus in Group One and 28.36% in plus in Group Two. On average, the participant in Group One reported an increase from 197.43° (SD 34.59°) to 239.59° (SD 46.13°). The participant in Group Two manifested an increase from 187.95° (SD 42.36°) to 241.25° (SD 51.79°). However, the dynamics of the observed amelioration did not differ statistically significantly between the study groups (*p* = 0.1428) (Table 7).

The Short Form (36-item) Health Survey (abbr. SF-36) was used in order to assess patients’ well-being after administration of stem cells. In line with the topic discussed here, four out of nine study areas were selected, i.e., focusing on the physical dimension of functioning of the studied patients.

The physical functioning changed significantly in both study subcohorts over 24 months of follow-up (respectively, *p* < 0.0001). In Group One, it ameliorated by 15.61%, while in Group Two, it deteriorated by 12.45%. The multi-factor analysis revealed that the dynamics of the changes did not differ statistically significantly between the subcohorts (*p* = 0.0903). At baseline, there was no statistically significant difference between them (*p* = 0.6900). In Group One, the most marked improvement was noticed 6 months after the SC administration (by 19.17%). After 24 months, it was not significantly different (Table 8).

The role limitations due to physical health were statistically significantly changeable in both study subcohorts over 24 months (respectively, *p* < 0.0001 and *p* = 0.0051). The relative change was 55.97% in plus in Group One and 2.66% in minus in Group Two. The dynamics of the phenomenon differed statistically significantly between the groups (*p* = 0.0022). A statistically meaningful difference in the assessment of role limitations was noticed between the subcohorts at baseline (*p* = 0.0007). On average, the discussed assessments amounted to 30.00% (SD = 37.01%) in Group One and 58.70% (SD = 31.63%) in Group Two. Surprisingly, the relative difference was 48.89% in favor of participants who had been administered SC earlier. In Group One, the largest benefit was felt 6 months after the procedure (by 61.60%), and in Group Two, after 12 months (by 25.26%). It was maintained in Group One, while in Group Two, it decreased (Table 9).

The assessment of pain severity—by using this particular research tool—did not fluctuate statistically significantly in both study subcohorts over 24 months (respectively, *p* = 0.0706 and *p* = 0.3537). The dynamics of the fluctuations did not vary between them (*p* = 0.4778), either. At baseline, no significant between-group difference was observed (*p* = 0.6393). It should be highlighted that the unchangeable, almost stable pain intensity throughout the 24-month period of observation does prove the protective, anti-inflammatory, analgesic, and trophic effect of stem cells on the affected hip joints (Table 10).

The evaluation of general health changed statistically significantly solely in Group One, not in Group Two (respectively, *p* < 0.0001 and *p* = 0.3498). In Group One, the relative improvement reached 32.08%. The dynamics were significantly varied in both subcohorts (*p* = 0.0016). At baseline, a statistically significant between-group difference was also noticed (*p* = 0.0133). On average, the study participants’ general health was assessed at 49.50% (SD = 12.54%) in Group One and 58.04% (SD = 19.35%) in Group Two. The relative difference, at baseline, was 14,71% in favor of Group Two. In Group One, the largest boost was observed 24 months after the SC administration (by 32.08%). It should be emphasized that the changes in Group Two admittedly were not significant, which means that the re-administration of SC does not worsen the general health appraisal in the studied patients (Table 11).

## 4. Discussion

The study introduces a novel approach to treating coxarthrosis through intra-articular administration of PBHSCs with CD34+ markers. The results demonstrate significant improvements in clinical outcomes and quality of life, underscoring the therapeutic potential of this innovative treatment method.

### 4.1. Pathophysiology of Osteoarthritis and Rationale for Stem Cell Therapy

OA is characterized by the breakdown of articular cartilage, subchondral bone remodeling, synovial inflammation, and osteophyte formation. These pathological changes result in pain, stiffness, and functional impairment [39]. Traditional treatments, including nonsteroidal anti-inflammatory drugs (NSAIDs), physical therapy, and surgical interventions, primarily aim to alleviate symptoms [40] but do not address the underlying degenerative processes.

### 4.2. Clinical Evidence Supporting Stem Cell Therapy in Hip Osteoarthritis

Stem cell therapy has emerged as a promising regenerative treatment for OA, aiming to repair damaged tissues, reduce inflammation, and restore joint function. Mesenchymal stem cells (MSCs), derived from sources such as bone marrow, adipose tissue, and synovial membrane, have been extensively studied for their chondrogenic differentiation potential and immunomodulatory properties [39,41]. The review concluded that intra-articular MSC therapy is safe with generally positive clinical outcomes. Clinical outcomes indicated improvements in pain relief and functional scores; however, the heterogeneity among studies and the lack of standardized protocols were noted as limitations. The evidence for orthobiologic treatments, primarily focusing on PRP injections, is limited, with insufficient data to support the use of mesenchymal stem cells or amniotic injectables for hip conditions [42].

A study by Emadedin et al. [43] focused on the intra-articular infusion of autologous bone marrow-derived MSCs in patients with different OA-affected joints, i.a., hip OA. The cohort consisted of eighteen patients who received BM-derived MSCs isolated and cultured. The study reported that this treatment was safe and therapeutically beneficial, suggesting continued studies with a larger size of patient groups and longer follow-up periods.

A systematic review and meta-analysis performed by Tian et al. [44] evaluated the relative efficacy and safety of MSCs for OA treatment. The analysis included 18 randomized controlled trials and concluded that MSCs were superior to placebo in relieving pain and improving function at the 12-month follow-up. Importantly, there were no significant differences in treatment-related adverse events between the MSC and placebo groups, emphasizing the safety profile of MSC therapy.

Another recent clinical study demonstrates that intra-articular injection of autologous adipose-derived MSCs in patients with hip OA yields positive preliminary results, though further long-term follow-up is needed to confirm the efficacy and sustainability of the technique [23].

The discussion in the area of stem cell therapy is also enriched by research on factors driving bone morphological changes in hip OA [45]. Analyzing synovium characteristics, synovial MSC differentiation potential, and synovial fluid composition highlighted the role of key genes and cytokines like CXCL8 and MMP9 in hypertrophic forms of the disease.

Several studies have confirmed clinical improvement following the intra-articular administration of hyaluronic acid, hyaluronic acid with gold nanoparticles, PRP, BMSCs, and ADCs [19,20,21,22,23,24,25]. With the exception of hyaluronic acid with gold nanoparticles, which demonstrated efficacy over a 24-month observation period [25], the effectiveness of the other injection types has been confirmed for durations ranging from 6 to 12 months [19,20,21,22,23,24]. In our study, the therapeutic effect of CD34+ cell therapy was confirmed after 24 months of follow-up. This allowed us to exclude the placebo effect, which, in the case of saline administration, may persist for up to six months [19].

Similar to previously published studies [19,20,21,22,23,24,25], we did not observe any early or long-term adverse effects following the administration of CD34+ cells. The therapeutic effectiveness of BMSC, ADC, and CD34+ cell therapy appears to be comparable [21,22,23,24]. Moreover, our findings support the observations of Tsitsilianos et al. [22], demonstrating that clinical improvement can be achieved regardless of the Kellgren–Lawrence (K-L) classification grade.

### 4.3. Comparative Analysis: PBHSCs CD34+ vs. MSCs

While MSCs have been the focal point of regenerative therapies for OA [46], our study explores the potential of PBHSCs CD34+. The application of PBHSCs CD34+ in the treatment of hip OA remains relatively unexplored. CD34+ cells are hematopoietic stem cells known for their high proliferative capacity and ability to promote angiogenesis. These properties may enhance tissue repair mechanisms, offering a distinct advantage over MSCs. The administration of PBHSCs CD34+ in our study resulted in significant improvements in pain reduction, joint function, and overall mobility. These findings suggest that PBHSCs may offer a viable alternative to current therapeutic strategies, including pharmacological interventions and surgical procedures such as THA.

### 4.4. Mechanisms of Action: Potential Benefits of PBHSCs CD34+

The therapeutic effects of PBHSCs CD34+ in OA may be attributed to several mechanisms [16,47]:

- Angiogenesis promotion: CD34+ cells have a well-documented role in promoting angiogenesis, which is crucial for delivering nutrients and oxygen to damaged tissues, facilitating repair and regeneration;
- Immunomodulation: similar to MSCs, PBHSCs may exert immunomodulatory effects, reducing synovial inflammation and altering the OA microenvironment to favor tissue repair;
- Paracrine Signaling: PBHSCs can secrete various growth factors and cytokines that may aid in cartilage repair and inhibit catabolic processes involved in OA progression.

But, explanation of the specific mechanism and influence on cartilage needs further studies [16].

### 4.5. Safety Profile

The safety of stem cell therapies is a paramount concern. Our study observed no severe adverse events associated with the intra-articular administration of PBHSCs CD34+. This finding aligns with previous studies on stem cell therapy [48].

### 4.6. Limitations and Future Directions

Our study has certain limitations. The sample size in our research significantly exceeds that of most previous studies involving BMSCs, ADCs, hyaluronic acid, or PRP [19,20,21,22,23,24,25]. Furthermore, statistical analysis revealed an appropriate statistical power, thus strengthening the validity of our findings. This is particularly relevant given our deliberate decision not to compare subgroups based on the severity of osteoarthritic changes according to the K-L classification.

Another limitation is the inclusion of rehabilitation, which is undoubtedly a factor influencing treatment outcomes in hip osteoarthritis [26]. Additionally, a crucial factor contributing to the sustained improvement in the range of motion is the joint mobilization we performed immediately after CD34+ cell administration. To the best of our knowledge, this therapeutic approach has not been previously reported in the available literature. Its implementation aimed to improve joint mobility, thereby increasing the contact surface between the femoral head and the acetabulum and optimizing exposure to CD34+ cells. In our opinion, increasing the load-bearing surface contact between the femoral head and the acetabulum is a key factor in stabilizing treatment outcomes over time. While dividing patients into groups with and without joint mobilization, as well as with and without rehabilitation, might seem methodologically appropriate, we believe that at the current level of knowledge, such a division would not provide additional valuable insights [26].

Despite the promising results, several limitations warrant further investigation:

- Sample size: our study group was relatively large compared to other studies on stem cells, but it is worth expanding the research to larger groups of patients and in multicenter settings. It is worth discussing the possibilities of further research funding to broaden its scope and enhance its clinical utility;
- Follow-up duration: while our studies with two-year follow-up demonstrated improvements in pain and function, focusing on longer follow-up periods is needed to determine the durability of the effects;
- Optimal dosage and frequency: further research should explore the most effective dosage and administration frequency of PBHSCs CD34+ for both therapeutic benefits and patient safety.

## 5. Conclusions

The conclusions for our research are the following:

1. Intra-articular administration of CD34+ cells significantly reduces pain and improves hip joint function, regardless of the severity of osteoarthritis according to the Kellgren–Lawrence classification, over a 24-month follow-up period;
2. The combination of CD34+ cell therapy with joint mobilization and rehabilitation allows for the postponement of hip arthroplasty by significantly improving patients’ quality of life over the 24-month follow-up period.

## Figures and Tables

**Table 1:** Baseline characteristics of the study cohort (n = 49 individuals = 95 hip joints).

Patient Characteristic	Statistical Parameter *
	n (%)/M (SD)
Gender:	
- Female	18 (36.73)
- Male	31 (63.27)
No. of stem cell administrations:	
- First (Group One)	72 (75.79)
- Second (Group Two)	23 (24.21)
Age (year)	61.05 (11.34)
Body weight (kg)	85.66 (16.03)
Body height (m)	1.73 (0.07)
Body Mass Index (kg∗m^−2^)	28.40 (4.46)

* For categorical variables, n—integer number, %—percentage. For continuous numerical traits, M—mean, SD—standard deviation. Missing data were pair-wise deleted.

**Table 2:** Assessment of pain according to HOOS (%) in the studied patients receiving stem cells over a 24-month follow-up by number of all-time injections (*p* = 0.1459 ^d^; statistical power > 0.9999).

Time Point	No. of Stem Cell Injections	Statistical Parameter *				*p* Value **
		M	SD	Me	Q_1_–Q_3_	
Baseline	First	74.27	23.48	83.75	52.50–95.00	=0.0001 ^a^
	Second	62.39	21.42	57.50	42.50–72.50	
After 6 weeks	First	73.62	21.72	75.00	55.00–97.50	
	Second	65.23	17.37	60.00	52.50–77.50	
After 3 months	First	75.82	23.60	80.00	50.00–97.50	
	Second	71.81	18.49	71.25	57.50–85.00	
After 6 months	First	81.21	18.79	83.75	68.75–100.00	
	Second	69.29	17.32	65.00	57.50–80.00	
After 12 months	First	82.13	18.44	87.50	75.00–100.00	
	Second	64.61	26.20	67.50	47.50–85.00	
After 24 months	First	81.16	20.02	87.50	72.50–100.00	<0.0001 ^b^ <0.0001 ^c^
	Second	57.95	21.70	52.50	37.50–75.00	

* M—mean, SD—standard deviation, Me—median, Q—quartiles. ** Controlled for the study subjects’ age, gender, and BMI. Levels of statistical significance are presented as follows: ^a^. between-group differences at baseline, ^b^. repeated measures for Group One, ^c^. repeated measures for Group Two, ^d^. between-group differences in the tested dynamics of a phenomenon in a multi-factor model, with repeated measures.

**Table 3:** Assessment of symptoms and stiffness according to HOOS (%) in the studied patients receiving stem cells over a 24-month follow-up by number of all-time injections (*p* = 0.0324 ^d^; statistical power = 0.9997).

Time Point	No. of Stem Cell Injections	Statistical Parameter *				*p* Value **
		M	SD	Me	Q_1_–Q_3_	
Baseline	First	71.04	23.13	75.00	57.50–90.00	=0.0004 ^a^
	Second	55.65	21.12	55.00	35.00–65.00	
After 6 weeks	First	67.84	25.36	70.00	50.00–90.00	
	Second	60.45	16.18	60.00	45.00–70.00	
After 3 months	First	72.91	23.03	75.00	50.00–100.00	
	Second	69.44	18.78	60.00	55.00–85.00	
After 6 months	First	75.55	21.64	77.50	60.00–95.00	
	Second	67.38	18.82	70.00	55.00–75.00	
After 12 months	First	75.37	22.92	77.50	65.00–95.00	
	Second	61.58	30.96	75.00	45.00–80.00	
After 24 months	First	76.71	21.55	80.00	65.00–95.00	<0.0001 ^b^ =0.0004 ^c^
	Second	53.18	20.77	55.00	40.00–70.00	

* M—mean, SD—standard deviation, Me—median, Q—quartiles. ** Controlled for the study subjects’ age, gender, and BMI. Levels of statistical significance are presented as follows: ^a^. between-group differences at baseline, ^b^. repeated measures for Group One, ^c^. repeated measures for Group Two, ^d^. between-group differences in the tested dynamics of a phenomenon in a multi-factor model, with repeated measures.

**Table 4:** Assessment of activities of daily living according to HOOS (%) in the studied patients receiving stem cells over a 24-month follow-up by number of all-time injections (*p* = 0.1872 ^d^; statistical power > 0.9999).

Time Point	No. of Stem Cell Injections	Statistical Parameter *				*p* Value **
		M	SD	Me	Q_1_–Q_3_	
Baseline	First	73.45	24.15	77.94	54.41–94.12	=0.0003 ^a^
	Second	63.17	21.19	57.35	47.06–80.88	
After 6 weeks	First	72.43	22.61	70.59	54.41–98.53	
	Second	65.91	20.18	63.97	50.00–88.24	
After 3 months	First	76.23	21.63	80.88	54.41–94.12	
	Second	74.43	18.19	77.21	52.94–86.76	
After 6 months	First	81.34	18.53	83.82	66.91–99.26	
	Second	70.73	17.22	72.06	55.88–82.35	
After 12 months	First	81.07	19.91	86.76	70.59–98.53	
	Second	69.89	27.84	70.59	57.35–92.65	
After 24 months	First	79.88	21.07	86.76	63.23–97.06	<0.0001 ^b^ =0.0002 ^c^
	Second	60.03	23.16	64.71	45.59–76.47	

* M—mean, SD—standard deviation, Me—median, Q—quartiles. ** Controlled for the study subjects’ age, gender, and BMI. Levels of statistical significance are presented as follows: ^a^. between-group differences at baseline, ^b^. repeated measures for Group One, ^c^. repeated measures for Group Two, ^d^. between-group differences in the tested dynamics of a phenomenon in a multi-factor model, with repeated measures.

**Table 5:** Assessment of sports and recreational activities according to HOOS (%) in the studied patients receiving stem cells over a 24-month follow-up by number of all-time injections (*p* = 0.0784 ^d^; statistical power > 0.9999).

Time Point	No. of Stem Cell Injections	Statistical Parameter *				*p* Value **
		M	SD	Me	Q_1_–Q_3_	
Baseline	First	66.23	28.10	75.00	37.50–93.75	=0.0003 ^a^
	Second	49.73	25.53	43.75	25.00–68.75	
After 6 weeks	First	60.07	28.12	56.25	43.75–81.25	
	Second	49.72	20.99	50.00	37.50–62.50	
After 3 months	First	68.41	24.13	68.75	50.00–93.75	
	Second	61.46	23.01	62.62	37.50–91.25	
After 6 months	First	72.36	23.51	75.00	50.00–98.75	
	Second	55.06	23.60	50.00	37.50–75.00	
After 12 months	First	69.33	24.62	75.00	50.00–93.75	
	Second	55.26	28.13	50.00	31.25–81.25	
After 24 months	First	71.49	26.48	75.00	56.25–100.00	<0.0001 ^b^ =0.0176 ^c^
	Second	43.18	25.07	50.00	12.50–62.50	

* M—mean, SD—standard deviation, Me—median, Q—quartiles. ** Controlled for the study subjects’ age, gender, and BMI. Levels of statistical significance are presented as follows: ^a^. between-group differences at baseline, ^b^. repeated measures for Group One, ^c^. repeated measures for Group Two, ^d^. between-group differences in the tested dynamics of a phenomenon in a multi-factor model, with repeated measures.

**Table 6:** Assessment of hip-related quality of life according to HOOS (%) in the studied patients receiving stem cells over a 24-month follow-up by number of all-time injections (*p* = 0.1104 ^d^; statistical power = 0.9997).

Time Point	No. of Stem Cell Injections	Statistical Parameter *				*p* Value **
		M	SD	Me	Q_1_–Q_3_	
Baseline	First	59.98	25.23	56.25	43.75–75.00	=0.0021 ^a^
	Second	42.39	26.45	43.75	18.75–56.25	
After 6 weeks	First	58.12	25.35	37.50	37.50–75.00	
	Second	44.60	18.23	43.75	21.35–50.00	
After 3 months	First	62.61	25.59	43.75	43.75–75.00	
	Second	52.78	18.84	51.12	43.75–62.50	
After 6 months	First	66.55	24.73	46.87	46.87–93.75	
	Second	44.94	21.80	43.75	31.25–56.25	
After 12 months	First	68.98	24.73	50.00	50.00–87.50	
	Second	48.36	26.17	73.75	25.00–68.75	
After 24 months	First	66.31	28.29	43.75	43.75–87.50	<0.0001 ^b^ =0.0015 ^c^
	Second	35.80	21.85	37.50	12.50–50.00	

* M—mean, SD—standard deviation, Me—median, Q—quartiles. ** Controlled for the study subjects’ age, gender, and BMI. Levels of statistical significance are presented as follows: ^a^. between-group differences at baseline, ^b^. repeated measures for Group One, ^c^. repeated measures for Group Two, ^d^. between-group differences in the tested dynamics of a phenomenon in a multi-factor model, with repeated measures.

**Table 7:** Ranges of movement (deg) in the studied patients’ hip joints after administering stem cells over a 24-month follow-up by number of all-time injections (*p* = 0.1428 ^d^; statistical power > 0.9999).

Time Point	No. of Stem Cell Injections	Statistical Parameter *				*p* Value **
		M	SD	Me	Q_1_–Q_3_	
Baseline	First	197.43	34.59	190.00	175.00–220.00	=0.2880 ^a^
	Second	187.95	42.36	172.50	165.00–205.00	
After 6 weeks	First	219.32	43.84	225.00	195.00–247.50	
	Second	204.76	46.73	195.00	160.00–245.00	
After 3 months	First	232.95	43.27	235.00	215.00–260.00	
	Second	225.00	48.65	240.00	210.00–265.00	
After 6 months	First	235.00	35.08	235.00	205.00–260.00	
	Second	242.89	41.00	245.00	230.00–275.00	
After 12 months	First	233.58	33.17	235.00	210.00–250.00	
	Second	243.23	26.39	250.00	225.00–260.00	
After 24 months	First	239.59	46.13	252.50	220.00–270.00	=0.0475 ^b^ =0.3689 ^c^
	Second	241.25	51.79	252.50	225.00–275.00	

* M—mean, SD—standard deviation, Me—median, Q—quartiles. ** Controlled for the study subjects’ age, gender, and BMI. Levels of statistical significance are presented as follows: ^a^. between-group differences at baseline, ^b^. repeated measures for Group One, ^c^. repeated measures for Group Two, ^d^. between-group differences in the tested dynamics of a phenomenon in a multi-factor model, with repeated measures.

**Table 8:** Assessment of physical functioning according to SF-36 (%) in the studied patients receiving stem cells over a 24-month follow-up by number of all-time injections (*p* = 0.0903 ^d^; statistical power > 0.9999).

Time Point	No. of Stem Cell Injections	Statistical Parameter *				*p* Value **
		M	SD	Me	Q_1_–Q_3_	
Baseline	First	54.89	25.28	50.00	35.00–75.00	=0.6900 ^a^
	Second	56.30	26.34	60.00	30.00–70.00	
After 6 weeks	First	58.63	27.04	50.00	40.00–85.00	
	Second	46.74	21.41	40.00	30.00–50.00	
After 3 months	First	58.28	21.94	60.00	45.00–75.00	
	Second	55.79	22.87	50.00	35.00–70.00	
After 6 months	First	65.41	23.82	65.00	45.00–85.00	
	Second	66.32	23.08	75.00	50.00–85.00	
After 12 months	First	58.48	27.45	55.00	35.00–85.00	
	Second	69.41	23.51	75.00	65.00–85.00	
After 24 months	First	63.46	25.76	65.00	40.00–90.00	<0.0001 ^b^ <0.0001 ^c^
	Second	49.29	30.69	55.00	20.00–60.00	

* M—mean, SD—standard deviation, Me—median, Q—quartiles. ** Controlled for the study subjects’ age, gender, and BMI. Levels of statistical significance are presented as follows: ^a^. between-group differences at baseline, ^b^. repeated measures for Group One, ^c^. repeated measures for Group Two, ^d^. between-group differences in the tested dynamics of a phenomenon in a multi-factor model, with repeated measures.

**Table 9:** Assessment of role limitations due to physical health according to SF-36 (%) in the studied patients receiving stem cells over a 24-month follow-up by number of all-time injections (*p* = 0.0022 ^d^; statistical power > 0.9999).

Time Point	No. of Stem Cell Injections	Statistical Parameter *				*p* Value **
		M	SD	Me	Q_1_–Q_3_	
Baseline	First	30.00	37.01	0.00	0.00–50.00	=0.0007 ^a^
	Second	58.70	31.63	50.00	25.00–75.00	
After 6 weeks	First	25.00	41.49	0.00	0.00–25.00	
	Second	32.61	43.59	0.00	0.00–100.00	
After 3 months	First	24.14	38.03	0.00	0.00–50.00	
	Second	34.21	38.38	25.00	0.00–75.00	
After 6 months	First	48.48	45.34	50.00	0.00–100.00	
	Second	63.16	41.97	75.00	25.00–100.00	
After 12 months	First	42.86	43.09	25.00	0.00–100.00	
	Second	73.53	39.00	100.00	50.00–100.00	
After 24 months	First	46.79	42.22	50.00	0.00–100.00	<0.0001 ^b^ =0.0051 ^c^
	Second	57.14	40.94	50.00	25.00–100.00	

* M—mean, SD—standard deviation, Me—median, Q—quartiles. ** Controlled for the study subjects’ age, gender, and BMI. Levels of statistical significance are presented as follows: ^a^. between-group differences at baseline, ^b^. repeated measures for Group One, ^c^. repeated measures for Group Two, ^d^. between-group differences in the tested dynamics of a phenomenon in a multi-factor model, with repeated measures.

**Table 10:** Assessment of pain according to SF-36 (%) in the studied patients receiving stem cells over a 24-month follow-up by number of all-time injections (*p* = 0.4778 ^d^; statistical power > 0.9999).

Time Point	No. of Stem Cell Injections	Statistical Parameter *				*p* Value **
		M	SD	Me	Q_1_–Q_3_	
Baseline	First	48.32	27.08	45.00	32.50–67.50	=0.6393 ^a^
	Second	51.96	29.31	45.00	22.50–70.00	
After 6 weeks	First	55.08	25.28	55.00	32.50–77.50	
	Second	50.43	16.83	45.00	32.50–65.00	
After 3 months	First	52.50	21.05	45.00	45.00–67.50	
	Second	60.53	20.20	55.00	55.00–67.50	
After 6 months	First	61.66	23.50	55.00	45.00–77.50	
	Second	60.79	25.38	55.00	35.00–77.50	
After 12 months	First	59.51	23.17	55.00	45.00–77.50	
	Second	63.23	22.48	67.50	45.00–77.50	
After 24 months	First	53.59	14.64	55.00	40.00–65.00	=0.0706 ^b^ =0.3537 ^c^
	Second	53.57	21.79	45.00	35.00–80.00	

* M—mean, SD—standard deviation, Me—median, Q—quartiles. ** Controlled for the study subjects’ age, gender, and BMI. Levels of statistical significance are presented as follows: ^a^. between-group differences at baseline, ^b^. repeated measures for Group One, ^c^. repeated measures for Group Two, ^d^. between-group differences in the tested dynamics of a phenomenon in a multi-factor model, with repeated measures.

**Table 11:** Evaluation of general health according to SF-36 (%) in the studied patients receiving stem cells over a 24-month follow-up by number of all-time injections (*p* = 0.0016 ^d^; statistical power > 0.9999).

Time Point	No. of Stem Cell Injections	Statistical Parameter *				*p* Value **
		M	SD	Me	Q_1_–Q_3_	
Baseline	First	49.50	12.54	50.00	40.00–60.00	=0.0133 ^a^
	Second	58.04	19.35	60.00	45.00–70.00	
After 6 weeks	First	53.23	12.61	52.50	50.00–60.00	
	Second	52.17	17.89	50.00	35.00–75.00	
After 3 months	First	51.29	10.07	50.00	50.00–60.00	
	Second	60.26	15.94	55.00	50.00–65.00	
After 6 months	First	52.95	11.23	52.50	45.00–60.00	
	Second	56.32	15.26	55.00	50.00–65.00	
After 12 months	First	50.36	13.10	50.00	45.00–55.00	
	Second	59.71	20.04	55.00	45.00–70.00	
After 24 months	First	65.38	26.04	75.00	50.00–75.00	<0.0001 ^b^ =0.3498 ^c^
	Second	53.57	37.80	50.00	25.00–100.00	

* M—mean, SD—standard deviation, Me—median, Q—quartiles. ** Controlled for the study subjects’ age, gender, and BMI. Levels of statistical significance are presented as follows: ^a^. between-group differences at baseline, ^b^. repeated measures for Group One, ^c^. repeated measures for Group Two, ^d^. between-group differences in the tested dynamics of a phenomenon in a multi-factor model, with repeated measures.

## Data Availability

The data presented in this study are available on request from the corresponding author.
